# Fascia training in patients undergoing allogeneic hematopoietic cell transplantation—a pilot study

**DOI:** 10.1007/s00520-022-07529-x

**Published:** 2022-12-16

**Authors:** Sandra Weigmann-Faßbender, Hanna Ulbricht, Marianne de Schultz, Christine Pawandenat, Desiree Kunadt, Michaela Wolff, Nadine Giesemann, Katja Prate, Johannes Schetelig, Martin Bornhäuser, Friedrich Stölzel, Nadja Knauthe, Friederike Stölzel

**Affiliations:** 1 Medical Faculty Carl Gustav Carus, Technical University Dresden, 01307 Dresden, Germany; 2 National Center for Tumor Diseases Dresden (NCT/UCC), Fetscherstraße 74, 01307 Dresden, Germany; 3 University Hospital Carl Gustav Carus Dresden, 01307 Dresden, Germany; 4 Technical University Dresden, 01069 Dresden, Germany

**Keywords:** Physical exercise, Oncology, Quality of life, Pain, Back flexibility

## Abstract

**Purpose:**

Patients undergoing allogeneic hematopoietic cell transplantation (alloHCT) spend many weeks of treatment in an isolated environment with little room for exercise. Feasibility of a daily-performed, unassisted fascia-training program and its effects on back and foot pain, back flexibility, and quality of life were investigated.

**Methods:**

Eighteen patients receiving alloHCT were randomized to an intervention (IG: *n* = 9; 60.7 ± 9.2 years) or control group (CG: *n* = 9; 54.0 ± 15.5 years) and assessed from 1 week before to 3 weeks after transplantation (t0–t3). CG received standard care physical therapy, IG performed additionally fascia training for the back and feet twice daily. Back and foot pain, back flexibility, muscle tone, and quality of life were assessed for both IG and CG at baseline and three timepoints after alloHCT.

**Results:**

Fascia-training program was well accepted. No increase in hematoma formation was observed. IG reported a trend towards reduction in back pain from pre- to post-intervention (*p* = .074), whereas CG showed a slight increase in back pain at t3 (*p* = .257). IG also improved back flexibility (− 1.79 ± 5.5 cm; *p* = .397) while CG declined (+ 2.71 ± 5.6 cm; *p* = .167). No differences between groups were found for muscle tone and no significant improvements in quality of life were reported at t3.

**Conclusion:**

Unassisted fascia training is feasible and safe for patients undergoing alloHCT. This pilot study suggests that fascia training has the potential to improve back flexibility and reduce back pain, and might be a valuable component for physical therapy in patients receiving alloHCT.

## Introduction

Allogeneic hematopoietic cell transplantation (alloHCT) is a potentially curative treatment in patients with hematologic malignancies, such as leukemia and lymphoma [1]. Prior to HCT, patients undergo conditioning therapy to reduce residual tumor burden and to allow engraftment of the donor’s hematopoiesis and immune system. Patients receiving alloHCT have a high risk for transplant-related mortality and a variety of physical and psychological side effects, such as life-threatening infections, graft-versus-host disease (GvHD), and impaired overall quality of life [2, 3]. Due to primary disease and consecutive treatment, patients are hospitalized for several weeks, oftentimes resulting in decreased levels of physical activity because of isolation during alloHCT [4]. Long periods of inactivity are associated with reductions in physical capacity, with muscle athrophy being one of its main consequences. Muscle atrophy in turn encourages foot and back pain as well as sensitivity disorders [5].

Current literature indicates that exercise is generally safe and feasible in patients with cancer and strong evidence for positive effects of exercise in this population was found [6–9]. These include decreased side effects, enhanced tolerance to treatment, improved functional outcomes, and, in some populations, improved survival outcomes. In the specific population of hematologic patients receiving alloHCT, feasibility and beneficial effects of exercise on prognosis and prevention of side effects have been well established in current studies [4, 10–15]. However, most studies focused on aerobic and strength training interventions [4, 10, 14, 15]. Over the last years, fascia rolls have been increasingly used in physiotherapy, rehabilitation, and exercise programs [16]. Fascia rolls have been shown to be effective in reducing back pain and increasing mobility [17, 18]. Current studies indicate positive effects of fascia training on the reduction of inflammation and endothelium functioning [19, 20].

The purpose of the present study was to examine feasibility and safety of a daily-performed, unassisted fascia training program in addition to daily physical therapy compared to a standard care procedure in patients receiving alloHCT. In addition, it was examined whether regular fascia training has the potential to reduce back and foot pain, and to improve back flexibility and muscle tone as well as quality of life in alloHCT-patients.

## Materials and methods

This prospective, monocentric pilot study is based on a randomized control group design with an ad hoc sample. The investigation was conducted from 1 week before to 3 weeks after HCT. The study was approved by the ethical review committee of the Technical University of Dresden (EK 169,042,019) and conducted in accordance with the Declaration of Helsinki.

### Participants

A total number of 194 patients underwent alloHCT at the University Hospital of Dresden from May 2019 to January 2021 (21 months). Inclusion criteria were (a) presence of hematological malignancy and receiving an alloHCT, (b) age ≥ 18 years, and (c) ability to provide informed consent and deliver study questionnaires. Exclusion criteria were (a) insufficient hematological capacity (platelets < 10 mmol/l), (b) acute pain, swelling or reddening of muscle tissue, and (c) motoric dysfunction or other comorbidities such as reduced stance and gait performance that impede participation in the exercise program. All patients had to sign informed consent.

**Fig. 1:**
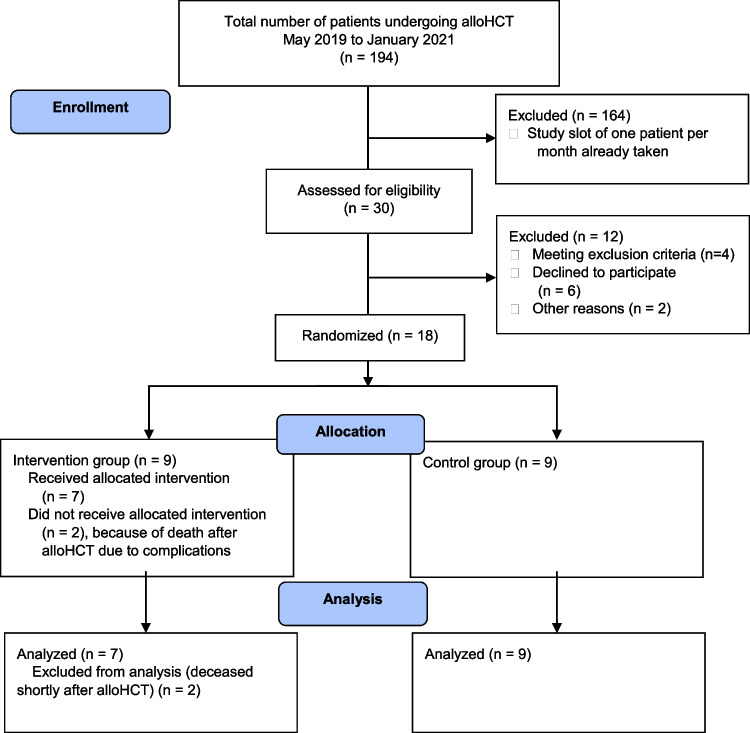
**a** Flowchart of study enrollment, allocation, and analysis

As one physical therapist attended all patients on the HCT ward and to maintain feasibility, it was determined to include only one patient at a time into the fascia-training study. Hospitalization for alloHCT patients roughly ranges from about 3 to 5 weeks, resulting in a mean of 21 patients that could complete the study consecutively within 21 months. As soon as one patient finished participation, the next patient entering the HCT ward would be informed about the study and, when consenting with participation, would then be randomized to an intervention group or a control group. Altogether, 30 patients were assessed for eligibility within this study frame. Six patients refused participation, four patients fulfilled at least one exclusion criterion at that time, and two patients were excluded due to participation in other clinical trials. Overall, 18 patients were randomized to an intervention group (IG, *n* = 9) or a control group (CG, *n* = 9). Both groups received standard care for alloHCT patients, which included daily physical therapy and comprises 15 to 45 min of supervised endurance and strength training using barbells, bicycle, and rowing machine ergometers, as well as mobilization, respiratory physiotherapy, and pain therapy depending on individual medical conditions. In addition, patients within the IG completed an unassisted daily fascia-training program (Fig. 1).

### Measures

Back and foot pain, back flexibility, and muscle tone as well as quality of life were assessed for both the intervention and control groups at baseline (t0) and three further points of measure (t1–t3). Back and foot pain were assessed based on the German version of Neuropathic Pain Symptom Inventory [21], using a numerical scale from 0 (no pain) to 10 (strongest pain). To assess back flexibility, a finger-floor distance test was conducted at each point of measure by a physical therapist with a measuring tape. Back muscle tone was determined by a physical therapist and was subjectively categorized into muscular hypertension, normal muscle tone, and muscular hypotension. Patients were asked to complete a questionnaire addressing quality of life developed by the European Organisation for Research and Treatment of Cancer [22]. The EORTC-QLQ-C30 consists of 30 questions from which global health status, five functional scores, three symptomatical scores, and six further single scores are calculated. Based on the character of the fascia-training program, the three scales physical functioning, fatigue, and global health status were analyzed.

### Fascia-training intervention

**Fig. 2:**
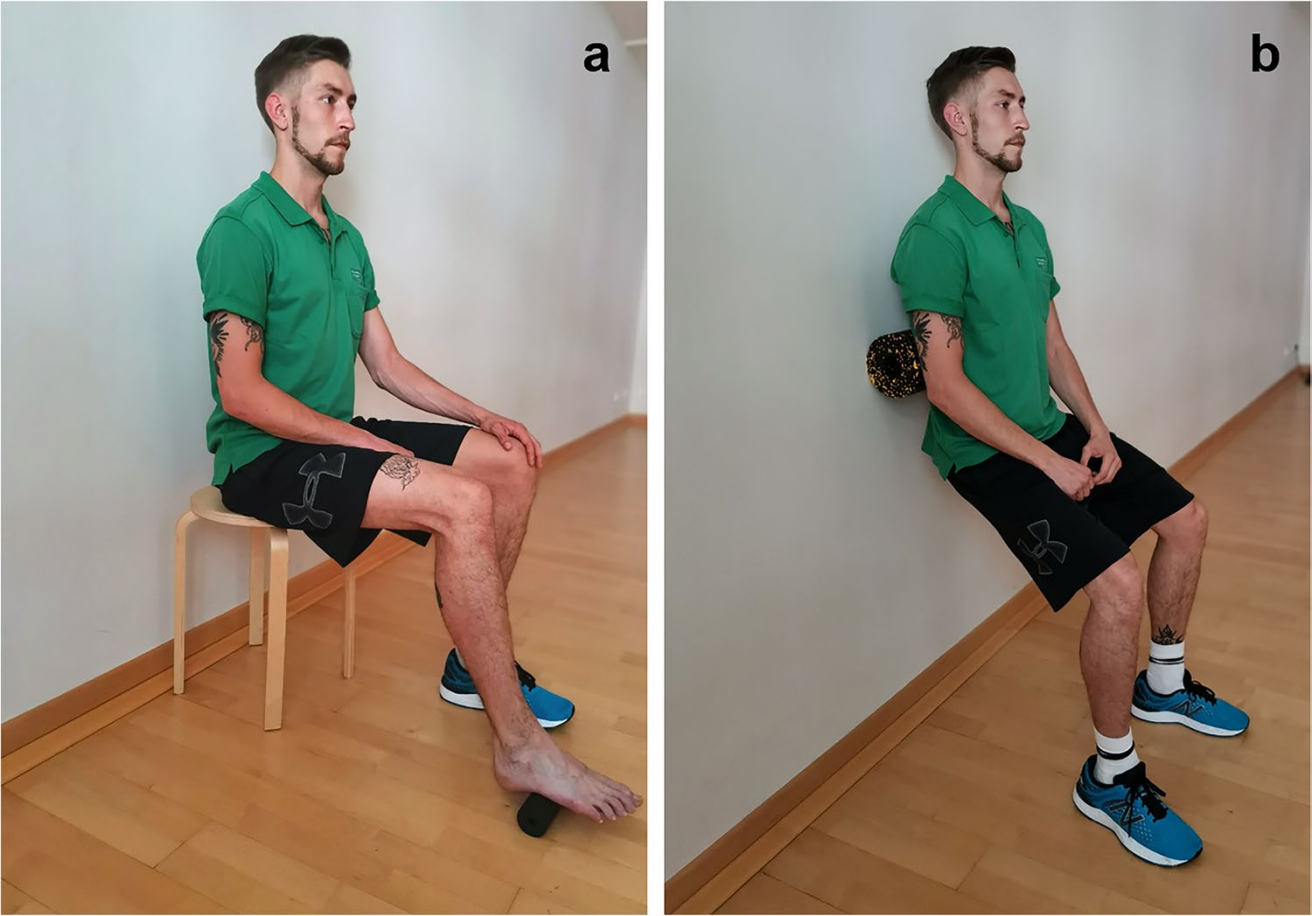
Fascia-training program exercise for **a** feet and **b** lower back; © University Hospital Dresden 2019

In addition to the standard care physical therapy, participants of the IG performed an unassisted fascia-training program twice daily in 5- to 7-min sessions, followed by 2 min of documentation. Beforehand, the fascia-training exercises were explained and demonstrated by a physical therapist. Exercises were conducted with very low tare weight to minimize the risk for patients with existing bleeding tendency (Fig. 2).

Training intervention consisted of two different exercises using fascia rolls Blackroll® standard fascia roll and Blackroll® Mini (BLACKROLL AG, Bottighofen, Schweiz) and Andhera fascia roll and BlackCat Mini fascia roll (#DoYourSports© GmbH, Berlin, Germany). The first exercise was performed sitting on the front edge of a chair. A small fascia roll was placed below the patient’s ball of the foot. The foot was then moved forward and backward for 30 to 60 s (Fig. 2a). This exercise was performed twice with each foot. The second exercise was performed in standing position with the fascia roll placed between the patient’s back and a wall. The patient then moved down slowly into a squat position according to his individual tolerance and subsequently returned to the starting position (Fig. 2b). This exercise was performed twice for 30 s each.

Contraindications and abort criteria for the fascia-training program were fever, strong pain that impedes physical exercise, nausea and dizziness, insufficient hematological capacity (platelets < 10 mmol/l), or acute bleeding signs.

### Statistical analysis

To evaluate effects of the fascia-training program from pre- (t0) to post- (t3) intervention, Wilcoxon tests were calculated for back and foot pain, back flexibility, muscle tone and the three scales of EORTC-QLQ-C30 global health, physical functioning, and fatigue. Statistical differences between IG and CG concerning changes in back and foot pain were calculated using Mann–Whitney *U* tests. Nonparametric tests were selected because of the small sample size and missing normal distribution. As this pilot study had exploratory character, a 5% significance threshold for all *p*-values was defined and data were not adjusted for multiple tests.

All statistical tests were processed using IBM SPSS Statistics, Version 28.0.1.0 (142) (Chicago, IL, USA). The low–high bar graph was created using GraphPad Prism, Version 5.01 for Windows (San Diego, CA, USA). Table data are presented as mean ± standard deviation. The low–high bar graphs depict lines as mean with bars ranging from minimum to maximum.

## Results

A total number of 18 patients were recruited to the present pilot study from May 2019 to January 2021 and were randomized to IG (*n* = 9, mean age: 60.7 ± 9.2 years, 7 male, 2 female) and CG (*n* = 9, mean age: 54.0 ± 15.5 years, 5 male, 4 female). The study participants underwent alloHCT due to malignant hematological neoplasms (Table 1). Two patients of the IG died shortly after alloHCT because of septic multiorgan failure in aplasia and were excluded from analysis. The mean duration of hospitalization for the IG was 29 days, whereas the mean duration for the CG was 32 days (range 24 to 45 days).

**Table 1:** Age, gender, and diagnoses of study participants

	Intervention (*n* = 9)	Control (*n* = 9)
Demographic characteristics		
Age, mean (SD)	60.7 (± 9.2) years	54.0 (± 15.5) years
Age, Min–Max	47–74 years	23–69 years
Gender, *N* females (%)	2 (22%)	4 (44%)
Diagnoses		
Acute myeloid leukemia	3	2
Myelodysplastic syndrome	3	2
Myeloproliferative neoplasms	2	2
Acute lymphatic leukemia		1
Acute biphenotypical leukemia	1	
Chronic lymphatic leukemia		1
Multiple myeloma		1

### Feasibilty

Patients of the IG (*n* = 7) were able to complete the prescribed daily fascia-training program with a mean of 24 (range 14–31) training days (83% of the amount calculated as mean value of days in hospital). Patients needed to interrupt the fascia-training program occasionally due to pain or discomfort, but all of them continued the intervention. No increases in hematoma formation and other serious events in conjunction with the training program were observed and patients reported acceptance and toleration of the fascia-training intervention.

### Foot and back pain

**Fig. 3:**
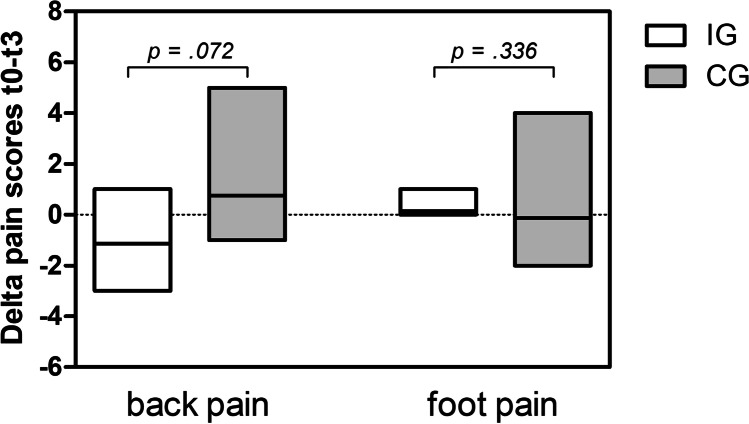
Changes in pain scores t0 to t3 in IG and CG

A tendency towards a reduction in levels of back pain from t0 to t3 was observed in IG (− 1.14 ± 1.5 points; *p* = 0.074). In contrast, CG showed a slight increase in levels of back pain from t0 to t3 (+ 0.75 ± 1.8 points; *p* = 0.257) (Fig. 3). Although there was an improvement in back pain in IG and a degration in CG from t0 to t3, no significant difference (*p* = 0.072) concerning the changes of back pain could be verified. Scores for foot pain did not change from t0 to t3 in both IG (*p* = 0.317) and CG (*p* = 0.713).

### Back flexibility and muscle tone

Back flexibility adaptation, measured by finger-floor distance test, was calculated as difference from t0 to t3. Two patients of CG were excluded from evaluation because they were not able to perform the exercises due to impairment by therapy. IG slightly improved back flexibility after daily fascia-training program by reduction of the finger-floor distance (− 1.79 ± 5.5 cm; *p* = 0.397), whereas CG showed a slight decline in back flexibility (+ 2.71 ± 5.6 cm; *p* = 0.167) (Table 2). However, the difference between IG and CG at t3 was not significant (*p* = 0.182).

**Table 2:** Back flexibility and quality of life at t0 and t3; *p < .05

Outcome parameters	Intervention (*n* = 7)			Control (*n* = 9)		
	t0	t3	*p*	t0	t3	*p*
Back flexibility						
Finger-floor-distance (cm)	17.3 ± 5.0	15.4 ± 8.3	.397	8.9 ± 7.3	11.3 ± 9.0	.167
Quality of life	(*n* = 7)			(*n* = 8)		
Global health status	52.4 ± 22.4	40.5 ± 19.5	.352	58.3 ± 22.7	42.7 ± 31.6	.262
Physical functioning	92.4 ± 12.4	68.6 ± 15.3	.041*	72.5 ± 26.5	53.3 ± 42.2	.326
Fatigue	39.7 ± 28.6	55.6 ± 20.3	.237	44.4 ± 32.0	54.2 ± 28.1	.575

In both groups, a muscular hypertension was observed at t0. After fascia-training program (t3), two patients of IG had normal muscle tone and five patients had light muscular hypertension. In CG, one patient had normal muscle tone, five patients had a muscular hypertension, and three patients could not be evaluated because they were not able to sit due to strong pain. Changes in muscle tone from pre (t0) to post (t3) intervention were not significant in both IG (*p* = 0.157) and CG (*p* = 0.317).

### Quality of life

Scales for physical functioning, fatigue, and global health status were analyzed from the EORTC-QLQ-C30 [22]. Global health status as well as physical functioning decreased and fatigue increased from t0 to t3 in both IG and CG (Table 2). The decrease of physical functioning in IG was significant (*p* = 0.041), but the mean score at t3 was still higher than that in CG.

## Discussion

The primary aim of this pilot study was to evaluate the feasibility and safety of a daily fascia-training program for patients undergoing alloHCT. Recent studies show that physical therapy and training is safe and effective for patients after alloHCT [4, 10, 15]. Due to conditioning, aplasia, immunosuppression, and therapy itself, these patients often have a reduced range of activity and are confined indoors for weeks. On this account, early in-room physical activation plays an important role to improve physical fitness, muscle strength, and quality of life and reduce fatigue of these patients. In addition to physical therapy as standard care procedure, a randomized group of alloHCT patients completed a daily fascia-training program using fascia rolls. In general, fascia training is feasible to improve back flexibilty and to reduce back pain [17–20]. However, to our knowledge, this is the first study investigating the feasibility and its potential effects of special fascia training in patients undergoing alloHCT.

The autonomously executed daily fascia-training program was well accepted and tolerated by the patients. Because of alloHCT and concomitant medical therapies, patients are at risk for bleeding and hematoma formation [3]. In the present pilot study, no increase in hematoma formation was observed. Patients were able to perform the fascia-training program on most of the prescribed days and no other serious events in conjunction with the training program were reported. Furthermore, no aggravation was observed concerning pain scores, back flexibility, and muscle tone.

Muscle atrophy caused by medication and prolonged inactivity frequently leads to back pain in patients after alloHCT. Previously published studies could verify positive effects of fascia training on back pain in healthy adults [17, 18]. In comparison, the IG of the present study reported a tendency towards a reduction in back pain from pre to post fascia-training intervention, whereas the CG showed a slight increase in back pain at t3. Daily fascia-training program might prevent an increase in back pain levels, although the differences between IG and CG were not significant.

Adhesion of fascias and concomitant reduction of flexibility result from immobility as well as inflammations and trauma [23]. Inactivity and rest could lead to thickenings due to an increase in shear strength, whereas body movement and vibration can liquefy sticky fascias [24]. The aim of a regular fascia training is rehydration of the connective tissue to achieve a greater range of motion (ROM), which is influenced by the flexibility of the lower back muscles and the posterior thigh muscles. Therefore, the finger-floor distance test was used to measure back flexibility before and after the fascia-training program in alloHCT patients. After 24 days of fascia training, the IG improved the finger-floor distance test by a mean of approximately 1.8 cm, whereas back flexibility of CG declined by a mean of approximately 2.7 cm.

In comparison to a study in healthy adults [25], patients of the present study used the fascia rolls only for exercises affecting the lower back muscles. Fascia roll exercises, targeting the posterior thigh muscles in addition to the lower back muscles, could provide additional effects.

Acknowledging that muscular hypertension is often associated with subjectively experienced muscle pain [26], the back muscle tone of alloHCT patients was evaluated. In this study, back muscle tone was subjectively categorized by a physical therapist into muscular hypertension, normal muscle tone, and muscular hypotension. IG and CG show similar results, but slightly lower back muscle tone in IG. However, the subjective assessment of this method might not be accurate enough to detect differences and other objective measures need to be considered for future studies.

Quality of life plays an important role in patients undergoing HCT. Investigations of 2800 stem cell–transplanted patients (allogeneic and autologous HCT) provide reference data for the subscales of EORTC-QLQ-C30 at different points of in- and outpatient treatment [2]. The greatest limitations in quality of life of these patients were reported during hospitalization after HCT. At t3, patients of the present study show similar scores for global health status to reference data at “hospitalization.” Physical functioning was higher in patients after fascia training (68.6 ± 15.3) than that in patients of CG (53.3 ± 42.2) and the reference group (54 ± 29) [2]. Concerning fatigue, patients of the present pilot study show lower mean scores at t3 (IG: 55.6 ± 20.3, and CG: 54.2 ± 28.1) than the reference sample (70 ± 30) during hospitalization. Besides positive effects in physical functioning, fascia training has the potential to reduce fatigue, emotional tension and anxiety [27, 28]. In addition to physical therapy as standard care procedure, fascia-training exercises might have a beneficial effect on physical functioning and fatigue in patients after alloHCT. However, the small patient number of the present study must be taken into consideration and other influencing factors cannot be ruled out. As inactivity is a problem not only during hospitalization but also in the subsequent time of follow-up care, a longer intervention as well as follow-up period should be planned for a larger investigation. This is enforced by fascial chronic graft-versus-host disease, a common and debilitating late complication of alloHCT, which might also be positively affected by maintaining fascial flexibility [29].

### Limitations

The present pilot study was conducted as a randomized controlled trial with two arms. Only 18 patients (9 per group) were recruited within 21 months. Due to feasibility reasons, only one patient at a time was able to be included in the study, thus not every patient had the chance to participate. The sample size was small and patients’ age, gender, and the underlying diseases were heterogeneous. Furthermore, other confounding variables cannot be ruled out. No clinically meaningful differences have been defined for the variables foot and back pain, back flexibility, and muscle tone, as well as for quality of life. This should be conducted in a consecutive study. The fascia training in this study was designed as a daily unsupervised program. Other studies show better results in supervised exercise interventions compared to home-based or unsupervised interventions [9]. In addition, training performance was only documented by the patients, training adherence and compliance were not otherwise monitored, which might have induced lower adherence. Furthermore, the two fascia-training exercises were very short with a daily training amount of 5 to 7 min and thus might have been insufficient to produce beneficial effects. An additional exercise for muscles of the posterior thigh region might also improve the overall outcome.

## Conclusion

Fascia-training exercises in addition to standard care physical therapy are feasible and safe for patients undergoing alloHCT. The present pilot study suggests that fascia-training exercises have the potential to improve back flexibility and reduce back pain and might be a valuable component for physical therapy in patients undergoing alloHCT. Low costs, low expenditure of time, and the opportunity of autonomous training also support fascia training to be integrated in alloHCT physical therapy standard care procedure.

The effect sizes and confidence intervals determined in this pilot study can be instrumental to plan larger investigations which aim at demonstrating improved flexibility, physical function, quality of life, and pain after fascia training in patients undergoing alloHCT.

## Data Availability

The datasets used and/or analyzed during the current study are available from the corresponding author on reasonable request.
